# Urinary continence after robot‐assisted radical prostatectomy with complete urethral preservation

**DOI:** 10.1002/bco2.70230

**Published:** 2026-05-25

**Authors:** Azka Yousaf, Ricardo Almeida‐Magana, Eoin Dineen, Tarek Al‐Hammouri, Zafer Tandogdu, Greg Shaw

**Affiliations:** ^1^ Division of Surgery and Interventional Science University College London London UK; ^2^ Department of Urology University College London Hospital NHS Foundation Trust London UK

**Keywords:** learning curve, oncological profile, robot‐assisted radical prostatectomy, urethral preservation, urinary incontinence

## Abstract

**Objectives:**

This study aimed to evaluate the functional, oncological and learning‐curve outcomes of the complete urethral preservation (CUP) technique during robot‐assisted radical prostatectomy in a large single‐centre cohort.

**Patients and Methods:**

We retrospectively analysed patients undergoing robot‐assisted radical prostatectomy (RARP) between June 2021 and August 2025 by a single high‐volume surgeon. Outcomes were compared by CUP status. Continence was assessed at early (3–6 months) and late (12 months) follow‐up, using a strict binary definition and with pad usage recorded separately. Oncological assessment included margin status (MS) and biochemical recurrence (BCR) over 3 years. Multivariable Cox regression assessed predictors of BCR. The learning curve was evaluated using LOESS‐based probability smoothing.

**Results:**

A total of 459 patients were included, with successful CUP achieved in 316 (68.8%). Early continence was higher in the CUP cohort than in non‐CUP patients (77% vs 67%, *p* = 0.02), while rates were similar at 12 months (83% vs. 80%, *p* = 0.74). CUP patients were significantly more likely to be pad‐free/security pad only at both timepoints (*p* = 0.019 and 0.012, respectively). Positive MS rates and locations were comparable between groups. CUP was not associated with increased BCR risk on multivariable analysis (HR 1.06, 95% CI 0.56–1.98; *p* = 0.90). CUP likelihood improved with experience.

**Conclusions:**

CUP is associated with improved early continence recovery and higher rates of pad‐free status following RARP, without compromising margin status or biochemical recurrence. These findings support CUP as a safe and reproducible continence‐preserving technique, with the likelihood of successful CUP increasing with surgical experience.

## 1 INTRODUCTION

Robot‐assisted radical prostatectomy (RARP) is a well‐established, potentially curative treatment option for localised prostate cancer (PC) but carries an inherent risk of erectile dysfunction and urinary incontinence (UI).^1^ Urinary continence is maintained by the external urethral sphincter and the surrounding connective and muscular supports of the membranous urethra, which provide both closure pressure and positional stability.^2^, ^3^ RARP can compromise these mechanisms through sphincter trauma, loss of urethral length and disruption of anterior and posterolateral support, contributing to early postoperative UI. While UI rates after 12 months can range between 4% and 31% of patients,^4^ recovery of continence within the early postoperative period is considerably poorer and highly variable.^2^ Early UI has major implications for patient quality of life and is associated with substantial financial costs both for patients and healthcare systems.^4^, ^5^

Numerous surgical modifications to RARP have been proposed to optimise early continence recovery after the procedure. These include reconstructive techniques to restore disrupted structures (anterior and posterior reconstruction^6^) and preservation‐based strategies aiming to maintain native urethral support mechanisms (hood technique,^7^ pre‐peritoneal space sparing (PSS)^8^). However, membranous urethral length (MUL) has been established as an independent predictor of continence outcomes;^9^, ^10^ therefore, strategies to preserve the length of urethra (bladder neck sparing^11^ and apical dissection^12^) have been proposed as an alternative method to prevent UI.

Our team has described the complete urethral preservation (CUP) technique^13^ which aims to maintain the length of the prostatic urethra and bladder neck during RARP. Data from our early series showed promising UI rates with low rates of positive surgical margins (PSM) and prostate specific antigen (PSA) biochemical recurrence (BCR) at 12 months.^13^ Here, we present an updated analysis of a single‐centre cohort comparing functional and oncological outcomes of patients in whom CUP was successfully performed versus patients with no CUP. We also present an analysis of the learning curve of the procedure.

## 2 METHODS

We retrospectively collected data for patients who underwent RARP at the University College London Hospital from June 2021 to August 2025. Surgeries were performed by a single high‐volume urological surgeon (G.S), using the da Vinci X/Xi® platform (Intuitive Surgical Inc., Sunnyvale, CA, USA). We have previously described the CUP technique along with a video.^13^ Briefly, CUP is performed via an anterior bladder neck approach. Following development of the pre‐peritoneal space, the bladder is incised proximal to the puboprostatic ligaments. Dissection proceeds along the avascular plane at the lateral vesico‐prostatic junction, with downward traction this looks like the ‘spine of an open book’, where blunt dissection with fenestrated bipolar forceps exposes the vertical urethral fibres. The urethra is mobilised intraprostatic distally down to the level of the verumontanum, where it is transected at its most distal point. The posterior urethra is carefully divided, and the ejaculatory ducts are separated from the urethral stump, while preserving the posterior urethral cuff. This technique maximises urethral length before completion of standard prostatectomy and vesicourethral anastomosis.

The CUP technique was attempted in all patients, except for those who had previous TURP, suspicion of prostate cancer invasion to bladder neck or periurethral tissues on MRI imaging. All patients who underwent surgery during the specified period were included. The quality of the surgery was graded to classify the success of whether CUP was achieved as per the following scoring matrix: No preservation achieved of the proximal urethra (0), end‐to‐end anastomosis but no urethral cuff (1), small/weak urethral cuff (2), strong/long urethral cuff (urethroplasty anastomosis) but shorter than full urethral length (3), complete urethral sparing (4). For analysis, patients with a score of 3 or 4 were classified as having successful CUP performed. When CUP was not possible, the case proceeded as a regular bladder neck sparing surgery or, if not feasible, then fishmouth reconstruction was performed. Furthermore, in patients with periurethral apical disease, retrograde (Schlomm) dissection was not performed, and the distal urethra, from verumontanum to the apical limit of the prostate, was sacrificed. These were scored as CUP Level 0–3, dependent on the total length of urethra spared.

Data collection was performed retrospectively using the electronic health records of patients, as part of a registered prospective audit within the quality assurance programme. The primary outcomes were assessment of continence at early (3–6 month) and late (12 month) timepoints. Secondary outcomes included assessment of oncological outcomes at last follow‐up and an assessment of the learning curve to perform the CUP procedure.

### 2.1 Continence Outcomes

A strict binary definition of continence was adopted; patients who reported any leakage at the specified visit would be classified as incontinent. Pad usage was also recorded to allow for an alternative assessment method as pad/leak free.

### 2.2 Oncological Outcomes

PSA data was collected at 12‐, 24‐ and 36‐months post‐surgery where available. Cases of biochemical persistence and/or recurrence were identified, defined as a PSA > 0.2 ng/mL, or if salvage radiotherapy was administered before this threshold. Histological factors such as margin positivity and location were also noted. A significant positive margin was defined as margin length >3 mm, or multifocal involvement.

### 2.3 Learning Curve

The learning curve for the technique was assessed by evaluating whether CUP was achieved utilising a binary definition of a quality of 3 and 4, against case number.

### 2.4 Statistical Analysis

Descriptive statistical analysis was performed for baseline, surgical and pathological variables. The primary outcome (UC) was analysed using the Fisher's exact test. Oncological outcomes were compared using Fisher's exact test and a survival analysis with a Cox regression model including known predictors of BCR (T stage, Grade group and margin status). Two‐sided *p*‐values of <0.05 were considered statistically significant. No adjustment for multiple testing was performed. Statistical analysis of the learning curve was performed using a LOESS‐based nonparametric probability smoothing model. Complete case analysis was performed as the rate of missing data was minimal.

All analyses were performed using R version 4.5.2 (R Foundation for Statistical Computing, Vienna, Austria).

## 3 RESULTS

A total of 459 patients underwent RARP between June 2021 and August 2025, with successful CUP achieved in 316 (68.8%). Baseline patient and operative characteristics are shown in Table 1.

**TABLE 1:** Baseline and operative characteristics by complete urethral preservation (CUP) status.

Characteristic	CUP^*1*^	No CUP^a^
Age (years)		
Median (Q1, Q3)	60 (56, 66)	61 (56, 66)
BMI (kg/m^2^)		
Median (Q1, Q3)	27.0 (25.0, 30.0)	28.0 (26.0, 31.0)
PSA (ng/mL)		
Median (Q1, Q3)	6.3 (4.9, 9.0)	7.7 (5.1, 12.0)
Cambridge prognostic group		
1	5 (1.6%)	4 (2.8%)
2	102 (32%)	37 (26%)
3	40 (13%)	15 (10%)
4	140 (44%)	59 (41%)
5	32 (10%)	28 (20%)
Membranous urethral length (mm)		
Median (Q1, Q3)	15.0 (12.0, 17.0)	14.0 (12.0, 16.0)
Missing	3	0
Operation time (mins)		
Median (Q1, Q3)	150 (120, 180)	150 (120, 180)
Console time (mins)		
Median (Q1, Q3)	120 (90, 150)	120 (90, 150)
Nerve spare status		
None	39 (12%)	27 (19%)
Unilateral	103 (32%)	39 (27%)
Bilateral	177 (55%)	77 (54%)
Blood loss (mL)		
Median (Q1, Q3)	200 (100, 300)	200 (120, 300)

^a^ *n* (%).

Distributions of ISUP grade group and pathological T stage were comparable between the CUP and non‐CUP cohorts, as seen in Table 2. Rates and locations of PSM did not differ significantly between groups.

**TABLE 2:** Pathological characteristics by complete urethral preservation (CUP) status.

Characteristic	CUP *N* = 319^a^	No CUP *N* = 143^a^	*p*‐value^b^
Pathological ISUP grade			
Grade 1	10 (3.1%)	4 (2.8%)	
Grade 2	197 (62%)	78 (55%)	
Grade 3	81 (25%)	37 (26%)	
Grade 4	17 (5.3%)	10 (7.0%)	
Grade 5	14 (4.4%)	14 (9.8%)	
Pathological T stage			
2	225 (71%)	95 (66%)	
3a	76 (24%)	41 (29%)	
3b	18 (5.6%)	7 (4.9%)	
Margin status			0.4
Negative	244 (76%)	105 (73%)	
Positive (not significant)	52 (16%)	22 (15%)	
Positive (significant)	23 (7.2%)	16 (11%)	
Location of positive margin^c^			0.4
Apex	13 (18%)	11 (29%)	
Base	5 (6.8%)	4 (11%)	
Circumferential	51 (69%)	22 (58%)	
Multifocal	5 (6.8%)	1 (2.6%)	

^a^ *n* (%).

^b^ Fisher's exact test.

^c^ Location shown only for patients with positive surgical margins.

**FIGURE 1:**
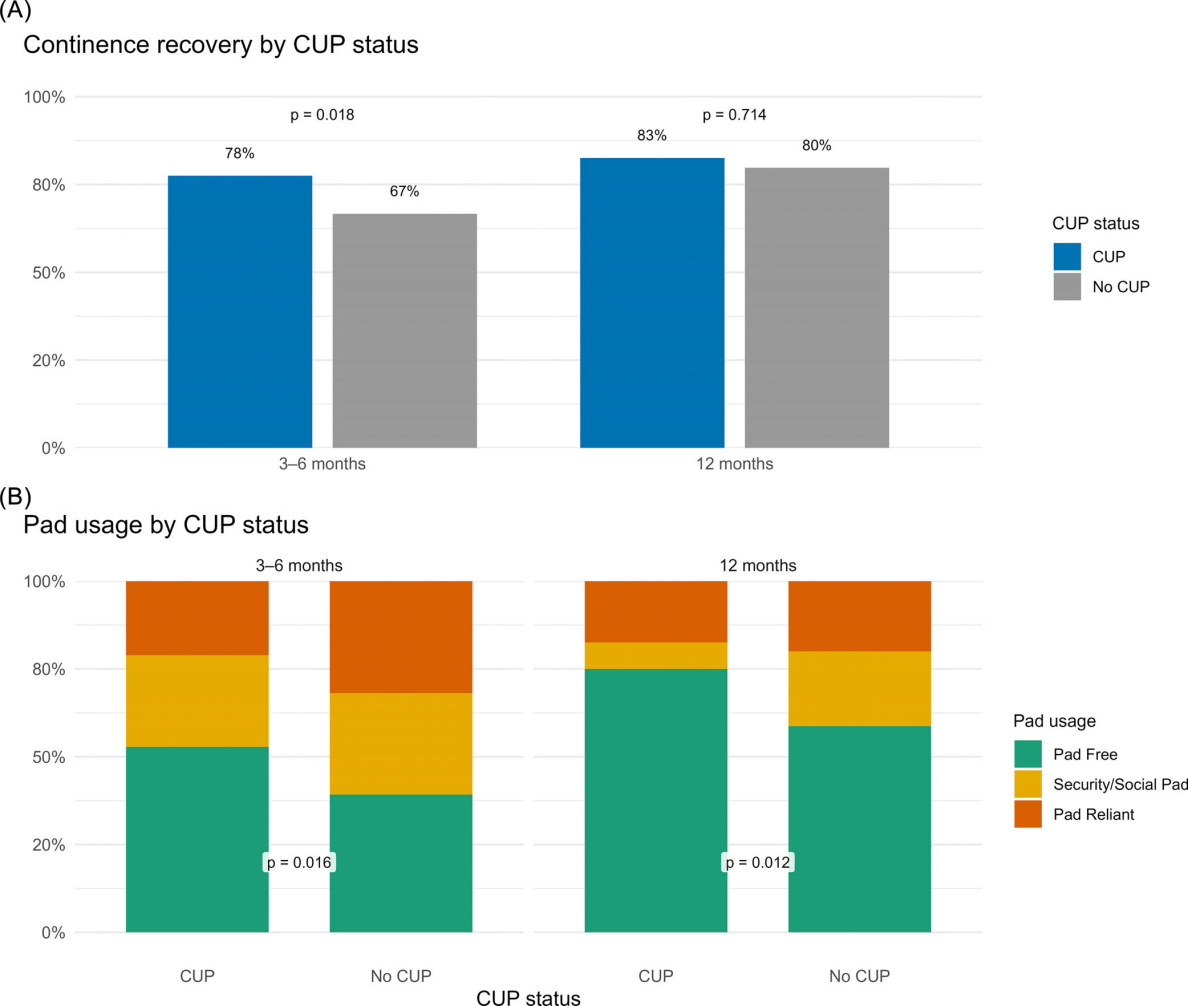
Continence recovery and pad usage by complete urethral preservation (CUP) status at early and late follow‐up. (A) Proportion of continent patients. (B) Distribution of pad usage categories. *P*‐values derived from Fisher's exact test.

Early continence was statistically significantly higher in the CUP cohort, versus the non‐CUP cohort as seen in Figure 1A, with continence rates of 78% and 67%, respectively (*p* = 0.02). This was not the case at 12 months; however, as continence rates show similarity with rates of 83% and 80%, respectively (*p*‐value = 0.71).

With regard to pad usage, depicted in Figure 1B, we found that the proportion of CUP patients at both early and late timepoints was using either no pad or one security/social pad compared with non‐CUP patients, and this was statistically significant (*p* = 0.02 at early, and 0.01 at late timepoints).

No patients experienced ureteric injury, nor required postoperative stenting/nephrostomy. Six patients overall developed urethral strictures requiring flexible cystoscopy and dilatation.

**FIGURE 2:**
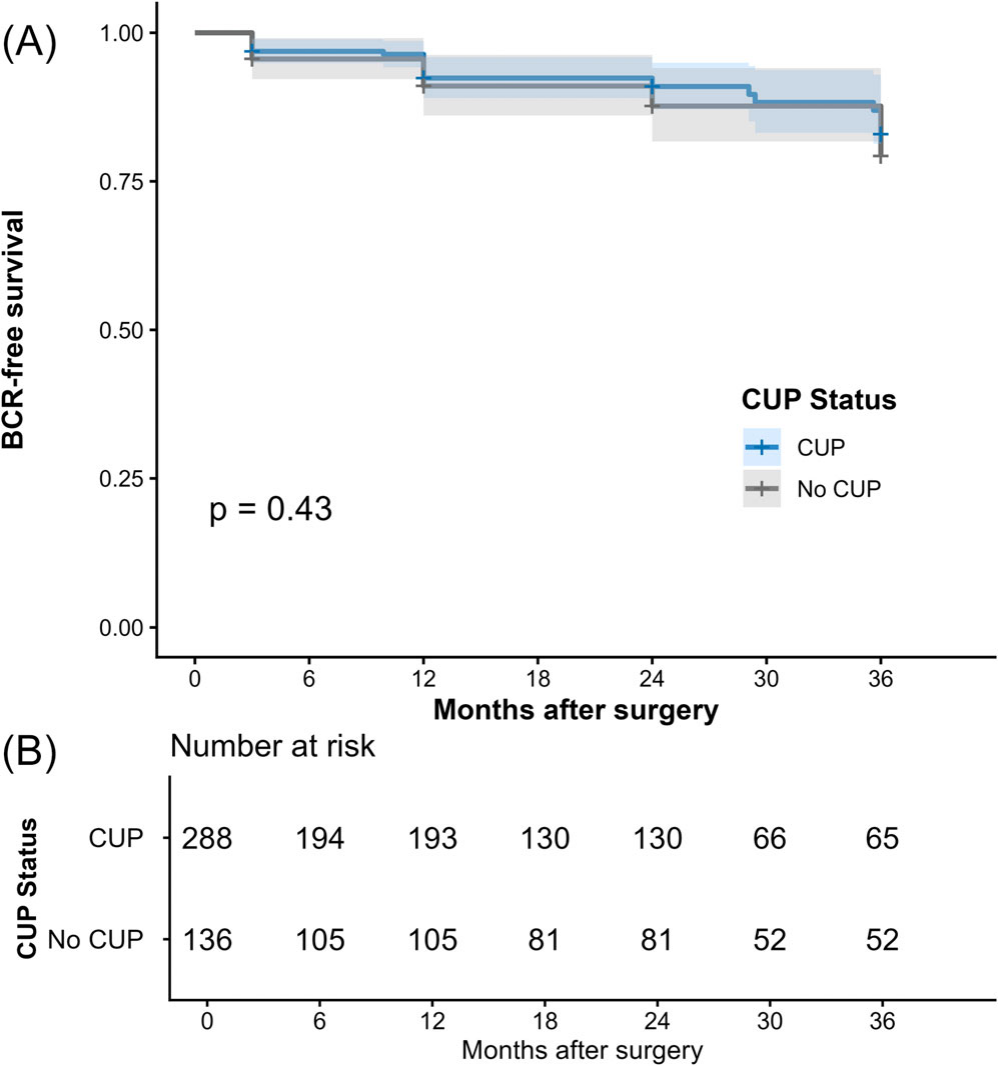
Kaplan–Meier estimates of biochemical recurrence–free survival, stratified by complete urethral preservation (CUP) status. (A) Biochemical recurrence–free survival curves with shaded areas representing 95% confidence intervals. (B) Number of patients at risk at each timepoint. *P*‐value derived from the log‐rank test.

Kaplan–Meier estimates of BCR–free survival stratified by CUP status are shown in Figure 2. Survival curves were comparable between groups, with no significant difference on log‐rank testing. Number‐at‐risk tables demonstrate steady attrition over time, with 65 patients in the CUP cohort and 52 in the non‐CUP cohort remaining under observation at 36 months.

Using multivariable Cox regression, as seen in Table 3, CUP was not associated with an increased risk of BCR; compared with patients without CUP, those undergoing CUP had a similar risk of BCR (HR 1.05, 95% CI 0.56–1.97; *p* = 0.90). Recurrence risk was driven by adverse pathological features rather than CUP status. Margin status and pathological tumour stage were the dominant predictors of BCR, with only significant positive margins and extraprostatic disease (pT3a–b) independently associated with increased recurrence risk. In contrast, nonsignificant positive margins were not associated with BCR, and CUP status itself was not an independent predictor of BCR.

**TABLE 3:** Multivariable Cox proportional hazards regression analysis of predictors of biochemical recurrence.

Characteristic	Hazard ratio	95% CI (lower)	95% CI (upper)	*p*‐value
**CUP status**				
No CUP	—	—		
CUP	1.05	0.56, 1.97	1.97	0.9
Margin status				
Negative	—	—		
Positive (not significant)	1.29	0.57, 2.91	2.91	0.5
Positive (significant)	**4.15**	1.93, 8.91	8.91	**<0.001**
Pathological ISUP grade				
1 and 2	—	—		
3	**2.05**	1.02, 4.10	4.10	**0.044**
4	1.51	0.49, 4.64	4.64	0.5
5	2.02	0.70, 5.81	5.81	0.2
Pathological T stage				
2	—	—		
3a	**2.55**	1.27, 5.11	5.11	**0.008**
3b	**5.47**	2.29, 13.1	13.1	**<0.001**

Abbreviations: CI, confidence interval; CUP, complete urethral preservation; HR, hazard ratio.

**FIGURE 3:**
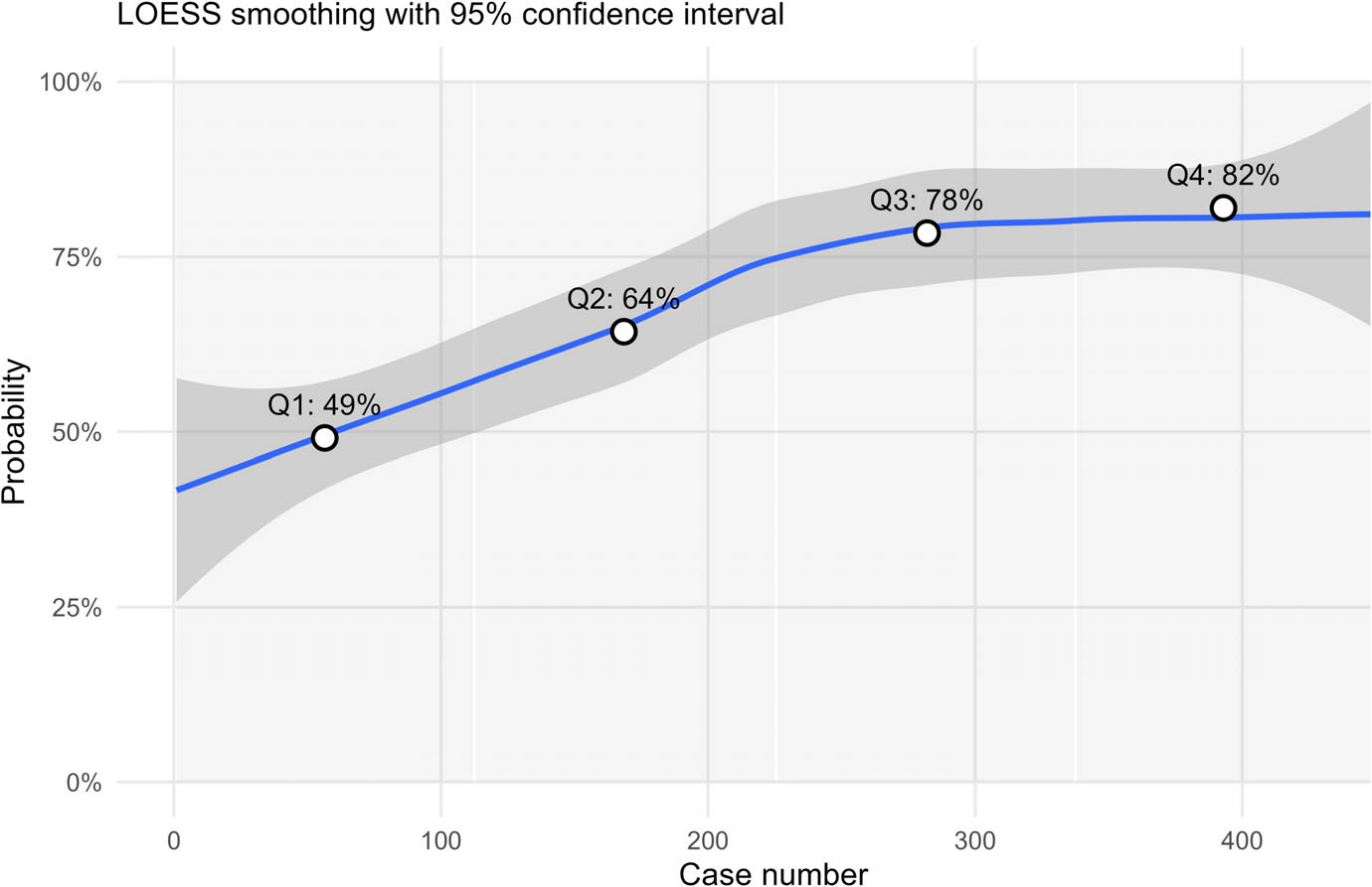
Probability of achieving complete urethral preservation (CUP) increases with case number. LOESS smoothing with 95% confidence interval.

Assessing the learning curve demonstrated a progressive improvement in CUP performance with increasing operative experience, as seen in Figure 3. CUP rates rose stepwise across experience quartiles. The LOESS‐smoothed probability curve showed a marked rise in the likelihood of achieving CUP during the first ~200 cases, with the curve beginning to plateau at approximately 300 cases. When CUP was not possible, the case proceeded as a regular bladder neck sparing surgery, except for 10 cases where fishmouth bladder neck reconstruction was performed.

## 4 DISCUSSION

Our findings suggest that when performed successfully, CUP is associated with a statistically significant higher rate of early continence recovery following RARP. Although continence rates converge by 12 months, the clear benefit at 3 months highlights an important advantage of this technique. Earlier continence recovery plays a role on improved quality of life, psychological wellbeing, and overall patient satisfaction after surgery.^14^, ^15^ Faster return to continence may also reduce the need for pads, community rehabilitation services, and later tertiary care, with associated cost savings that are particularly relevant for publicly funded healthcare systems such as the NHS.^5^

Variation in the definition of continence across the literature remains a challenge to allow for meaningful comparisons of functional outcomes.^4^, ^16^, ^17^ Our definition of continence was strict, classifying any instance of urinary leakage as incontinence. This conservative approach follows suggestions made by other authors^18^, ^19^ to avoid overestimating success and help standardise the reporting of post‐prostatectomy outcomes. To further standardise UI outcomes, we are now collecting validated patient‐reported outcome measures, such as the EPIC‐26^20^ or ICIQ‐SF questionnaires.^21^ Assessment of pad usage can provide a meaningful insight into how continence recovery affects daily life. In our cohort, CUP patients were significantly more likely to use no pads or only a single security pad at both early and late timepoints, suggesting a smoother and more confident transition to social and physical normality.

Continence‐sparing techniques can be broadly divided into preservation‐based strategies (e.g. bladder neck preservation and apical urethral preservation) and reconstructive techniques (e.g. posterior reconstruction). Systematic reviews, such as Ippoliti et al.,^22^ show that while many of these approaches offer some benefit, maintaining urethral length appears to have one of the most consistent effects, with each millimetre potentially translating into measurable continence advantage.^16^, ^23^, ^24^ By maximally preserving MUL, CUP enhances urethral coaptation and pressure transmission, while enabling a more anatomical vesicourethral anastomosis, or as the case with CUP, urethral‐urethral anastomosis. It therefore falls within the remit of a preservation‐based approach, and noticeably the pattern of earlier continence recovery as seen with our CUP analysis, closely mirrors that reported for PSS‐RARP. Comparatively however, as CUP is performed via an anterior approach, it affords improved access and visualisation of key anatomical planes, which may facilitate more reliable urethral preservation than compared with PSS‐RARP. In a recent systematic review and meta‐analysis by Ficarra et al.,^25^ PSS‐RARP was associated with a significant early continence advantage at 3 months (pooled OR 4.71), while analyses restricted to randomised trials suggested that this benefit attenuates and is no longer statistically significant by 12 months (OR 1.36). In keeping with this pattern, while overall continence rates converged by 12 months in our cohort, CUP conferred superior early continence recovery and, importantly, a higher likelihood of pad‐free status at late follow‐up.

A key and important consideration with alteration to surgical techniques is that of oncological control. A recent meta‐analysis by Xiong et al.^10^ reported that urethral‐length preservation did not increase overall PSM rates, supporting the biological plausibility and oncological safety of this preservation‐based approach. In concordance, our results also suggest that utilisation of the CUP technique does not compromise oncological control. Our multivariable Cox regression analysis demonstrated that CUP status was not associated with an increased risk of BCR, consistent with previous reports of MUL‐conserving and urethral‐preserving approaches, which have similarly shown oncological equivalence to standard RARP.^26^, ^27^ Margin positivity rates and the distribution of margin sites, most notably the apex and base, were similar between our groups, again supporting the oncological non‐inferiority of CUP.

Concerns regarding PSM have been reported for other continence‐preserving techniques, particularly PSS‐RARP.^28^, ^29^ In a meta‐analysis by Tai et al.,^30^ PSS‐RARP was associated with a significantly higher overall PSM rate compared with conventional RARP, with subgroup analyses demonstrating increased PSM rates in randomised trials and at anterior margin sites. Importantly, in our cohort, CUP was not associated with increased overall margin positivity, suggesting that CUP can be achieved without an apparent trade‐off in oncological safety. Our assessment of the learning curve showed that the likelihood of successfully achieving CUP increased with case number, reaching a plateau around case 300.

This study has several limitations. It reflects a single‐centre, single‐surgeon experience, which may limit generalisability, and the retrospective design introduces an inherent risk of selection and documentation bias. Oncological follow‐up was limited to 3 years, and event numbers remain relatively small in some pathological subgroups. Longer prospective follow‐up, multi‐surgeon evaluation and inclusion of validated PROMs will be important next steps, which our team is planning to undertake in the near future.

## 5 CONCLUSION

In summary, CUP aids the recovery of urinary continence without compromising oncological safety. The functional improvements observed are most pronounced during the early postoperative period; however, significant improvements in pad‐free/leak‐free rates were seen at 12 months. Despite promising early findings, a planned multicentre randomised controlled trial will provide definitive conclusions as to the benefits of CUP for patients.

## AUTHOR CONTRIBUTIONS

Study design, data collection, data analysis and manuscript preparation: Azka Yousaf. Study design, data analysis and manuscript review: Ricardo Almeida‐Magana. Study design and manuscript review: Eoin Dineen, Tarek Al‐Hammouri, Zafer Tandogdu, Greg Shaw.

## CONFLICT OF INTEREST STATEMENT

Ricardo Almeida‐Magana receives a salary from the National Institute for Health and Care Research (NIHR; RFPB PB‐PG‐1216‐20013), JP Moulton Charitable Foundation 17/0043 and Prostate Cancer UK MA‐CT20‐011 and has received support from SamanTree Medical SA to attend a conference in 2025 (NARUS 2025).

Eoin Dinneen: Received a salary from NIHR RFPB PB‐PG‐1216‐20 013 and from JP Moulton Charitable Foundation 17/0043 while working as the NeuroSAFE PROOF clinical research fellow.

Greg Shaw: Received honoraria from Astellas, Ipsen, and Janssen. He has previously acted as a paid medical consultant for Angle plc. He has received financial support for conference attendance from SamanTree Medical SA. He is currently a medical consultant for Samantree and holds shares in Samantree Medical SA. All other authors declare no conflict of interest in relation to this publication.
